# *Tityus serrulatus* envenoming in non-obese diabetic mice: a risk factor for severity

**DOI:** 10.1186/s40409-016-0081-8

**Published:** 2016-09-17

**Authors:** Guilherme Honda de Oliveira, Felipe Augusto Cerni, Iara Aimê Cardoso, Eliane Candiani Arantes, Manuela Berto Pucca

**Affiliations:** 1 Department of Physics and Chemistry, School of Pharmaceutical Sciences of Ribeirão Preto, University of São Paulo (USP), Av. do Café, s/n, 14040-903 Ribeirão Preto, SP Brazil; 2 Medical School of Roraima, Federal University of Roraima (UFRR), Av. Capitão Ene Garcez, 2413, Boa Vista, RR 69310-000 Brazil

**Keywords:** *Tityus serrulatus*, Diabetes mellitus, Scorpion venom, NOD mice, Glycemia

## Abstract

**Background:**

In Brazil, accidents with venomous animals are considered a public health problem. *Tityus serrulatus* (Ts), popularly known as the yellow scorpion, is most frequently responsible for the severe accidents in the country. Ts envenoming can cause several signs and symptoms classified according to their clinical manifestations as mild, moderate or severe. Furthermore, the victims usually present biochemical alterations, including hyperglycemia. Nevertheless, Ts envenoming and its induced hyperglycemia were never studied or documented in a patient with diabetes mellitus (DM). Therefore, this is the first study to evaluate the glycemia during Ts envenoming using a diabetic animal model (NOD, non-obese diabetic).

**Methods:**

Female mice (BALB/c or NOD) were challenged with a non-lethal dose of Ts venom. Blood glucose level was measured (tail blood using a glucose meter) over a 24-h period. The total glycosylated hemoglobin (HbA1c) levels were measured 30 days after Ts venom injection. Moreover, the insulin levels were analyzed at the glycemia peak.

**Results:**

The results demonstrated that the envenomed NOD animals presented a significant increase of glycemia, glycosylated hemoglobin (HbA1c) and insulin levels compared to the envenomed BALB/c control group, corroborating that DM victims present great risk of developing severe envenoming. Moreover, the envenomed NOD animals presented highest risk of death and sequelae.

**Conclusions:**

This study demonstrated that the diabetic victims stung by Ts scorpion should be always considered a risk group for scorpion envenoming severity.

**Electronic supplementary material:**

The online version of this article (doi:10.1186/s40409-016-0081-8) contains supplementary material, which is available to authorized users.

## Background

In Brazil, accidents involving venomous animals are considered a public health problem. *Tityus serrulatus* (Ts), popularly known as the yellow scorpion, is most frequently responsible for these accidents. During the period from 2000 to 2015, 727,113 cases of scorpion envenoming were reported in Brazil, with 1026 deaths and a mortality rate of 0.14 % [1–4].

Envenoming by Ts can cause several signs and symptoms according to not only the content of venom, but also the victim’s body weight, the blood-brain-barrier permeability, sex, health conditions and sting location. The mild envenoming is characterized by an intense local pain and possible paresthesia. Moderate envenoming manifests through local pain as well as nausea, sweating, vomiting, tachycardia, tachypnea and increasing of blood pressure. Severe envenoming presents the same symptoms of moderate followed by agitation and exhaustion, abdominal pain, stiffness and muscle spasms, convulsions, fever, dehydration, cardiac arrhythmias, heart failure and even coma [5–11]. Moreover, biochemical alterations are also observed during Ts envenoming such as hyperglycemia [6, 12–15].

Hyperglycemia is a sign constantly observed in diabetic individuals. Diabetes mellitus (DM) currently affects about 314 million people worldwide. Solely in 2012, it was responsible for 1.5 million deaths according to the World Health Organization (WHO). Currently, based on the etiology of the disease, DM can be classified into two types: type 1 and type 2 [16]. Although the pathologies have different origins, both have similar signs and symptoms such as hyperglycemia, polyuria, polyphagia, polydipsia, unexplained weight loss and may also include foot pain, blurred vision, frequent infections and even coma [17, 18].

The type 1 DM, or insulin-dependent DM, has a genetic etiology with the most common type being among children and juveniles. It is an autoimmune disease in which self-reactive T cells induce the B cell production of specific antibodies against beta cells of the islets of Langerhans. This autoimmune mechanism results in the destruction of these cells and consequently the decrease of insulin production [19, 20]. On the other hand, type 2 DM is the most common type of diabetes (accounts for 90 %). It is considered a chronic metabolic disorder and has been characterized by impaired insulin action and/or abnormal insulin secretion and eventual pancreatic beta-cell failure. An early abnormality in the disease is insulin resistance, which is the key linking factor for the metabolic syndrome disease cluster of glucose intolerance, hypertension and dyslipidemia [21–23].

The model employing non-obese diabetic (NOD) animals is useful for type 1 diabetes, and presents an autoimmune genetic disease where T cells (CD4^+^andCD8^+^) become self-reactive to pancreatic islets, resulting in inflammation during the first 3 to 4 weeks of life. However, only after mice reach 4 to 6 months of age does it become possible to verify insulitis, which results in insulin deficiency and therefore the clinical signs of diabetes [24, 25]. Thus, based on the Ts venom-induced hyperglycemia and the high incidence of diabetic patients, this is the first study to investigate the Ts envenoming complications in DM individuals, using NOD mice as the experimental model.

## Methods

### *T. serrulatus* venom

The Ts scorpions, collected from the Ribeirão Preto region, were kept in the Serpentarium of the Medical School of RibeirãoPreto (FMRP/USP). The venom extraction from 145 scorpions was performed using the telson electrical stimulation method ─ 12 mV [26]. After extraction, the pooled venom was desiccated and stored at –20 °C. The use of Ts venom was approved by the Genetic Patrimony Management Board (CGEN/MMA), through the Access and Shipment Component of Genetic Heritage for scientific research purposes (number 010174/2014-1).

### Mass assessment of the venom of *T. serrulatus*

The desiccated venom was dispersed in 1 mL of ultrapure water and centrifuged at 10,015 × *g*, 4 °C for 10 min, and the supernatant was stored at 4 °C. The pellet was resuspended using the same conditions. The total supernatant (2 mL) resulted in soluble pooled venom without the presence of mucus.

The mass of soluble pooled venom was estimated by absorbance readings at 280 nm using the NanoDrop spectrophotometer 2000 (Thermo Scientific, USA) and the extinction coefficient of the soluble venom [27]:

$$ \varepsilon \frac{\ 1\ \mathrm{mg}/\mathrm{mL}}{280\ \mathrm{nm}}=1.65 $$

### Tricine-SDS-PAGE

The venom was analyzed using tricine sodium dodecyl sulfate polyacrylamide gel electrophoresis (Tricine-SDS-PAGE) according to the method used for ultra-low-mass proteins [28]. The 16.5 % separating gel used was overlaid by a 5 % stacking gel. Samples consist of different masses of Ts venom (10, 20 and 30 μg) and the molecular mass marker (M-3546, Sigma-Aldrich®, USA). The gel was stained with Coomassie Blue plus one PhastGel® R-350 (GE Healthcare, Sweden) and destained with 10 % acetic acid (V/V).

### Animals

Females of BALB/c and NOD lineage (18–25 g) were obtained from the biotherium of the School of Pharmaceutical Sciences of Ribeirão Preto (FCFRP/USP) and the biotherium of the Ribeirão Preto Medical School (FMRP/USP), respectively. The animals were kept in cages with filters in an air-conditioned environment (23 ± 1 °C, 55 ± 5 % humidity) until the blood glucose values of NOD animals became significantly higher than those of the controls (BALB/c), indicating the hyperglycemia characteristic of the diabetic state (15 weeks old). Food and water were provided *ad libitum*. Mouse experimental models are in accordance with the Ethical Principles in Animal Experimentation under the license number 13.1.372.53.0.

### Basal blood glucose levels and *T. serrulatus* venom dose

The basal glucose levels of BALB/c and NOD mice were measured in tail blood using a glucose meter (One Touch Ultra®, Lifescan, USA). The dose of Ts venom capable of inducing hyperglycemia in mice (BALB/c and NOD) was also adjusted. The doses of 1 mg/kg and 0.5 mg/kg were tested in the different mouse species.

### Kinetic assay of glucose induced by *T. serrulatus* venom

Groups of female BALB/c or NOD mice (18–25 g, *n* = 4) were challenged with a non-lethal dose of Ts venom (0.5 mg/kg) using subcutaneous injection (similar to scorpion sting site), diluted in sterile physiological solution (0.9 % W/V of NaCl) in a final volume of 0.2 mL. Control groups received only sterile physiological solution. Glucose was measured in tail blood using a glucose meter (One Touch Ultra®, Lifescan, USA). Blood glucose was measured over a 24-h period (at 0, 1, 2, 3, 4, 5, 6, 12 and 24 h).

### Glycosylated hemoglobin (HbA1c) induced by *T. serrulatus* venom

Groups of female BALB/c or NOD mice (18–25 g, *n* = 4) were challenged with a non-lethal dose of Ts venom (0.5 mg/kg) using subcutaneous injection (similar to scorpion sting site), diluted in sterile physiological solution (0.9 % W/V of NaCl) in a final volume of 0.2 mL. Control groups received only sterile physiological solution. After 30 days (to reflect mean glycemia for the previous 30 days), 0.5 mL of blood from the retro-orbital cavity was collected in heparinized tubes under intraperitoneal anesthesia: ketamine 60 mg/kg (Dopalen, Agripands Brasil Ltda®, Brazil) and xylazine 8 mg kg (Rompun, Bayer Animal Health®, Brazil). The blood was centrifuged at 10,000 rpm for ten minutes, at 4 °C, to obtain the plasma. The measurement of total glycosylated hemoglobin (HbA1c) levels was performed according to the manufacturer’s instructions (Doles®, Brazil).

### Insulin levels induced by *T. serrulatus* venom

Groups of female BALB/c or NOD mice (18–25 g, *n* = 4) were challenged with a non-lethal dose of Ts venom (0.5 mg/kg) using subcutaneous injection (similar to scorpion sting site), diluted in sterile physiological solution (0.9 % W/V of NaCl) in a final volume of 0.2 mL. Control groups received only sterile physiological solution. After reaching the hyperglycemia peak (1 h after envenomation), 0.5 mL of blood from the retro-orbital cavity was collected in heparinized tubes under anesthesia. The blood was centrifuged at 10,000 rpm for 10 min, at 4 °C, to obtain the plasma. The insulin assay was performed using the immunoassay method according to the manufacturer’s instructions (Ultra Sensitive Mouse Insulin ELISA kit, Crystal Chem, USA).

## Results

### *T. serrulatus* venom mass and electrophoresis

The pooled venom obtained from 145 Ts scorpions resulted in 21 mg of soluble venom, a median of 0.14 mg per scorpion.

**Fig. 1:**
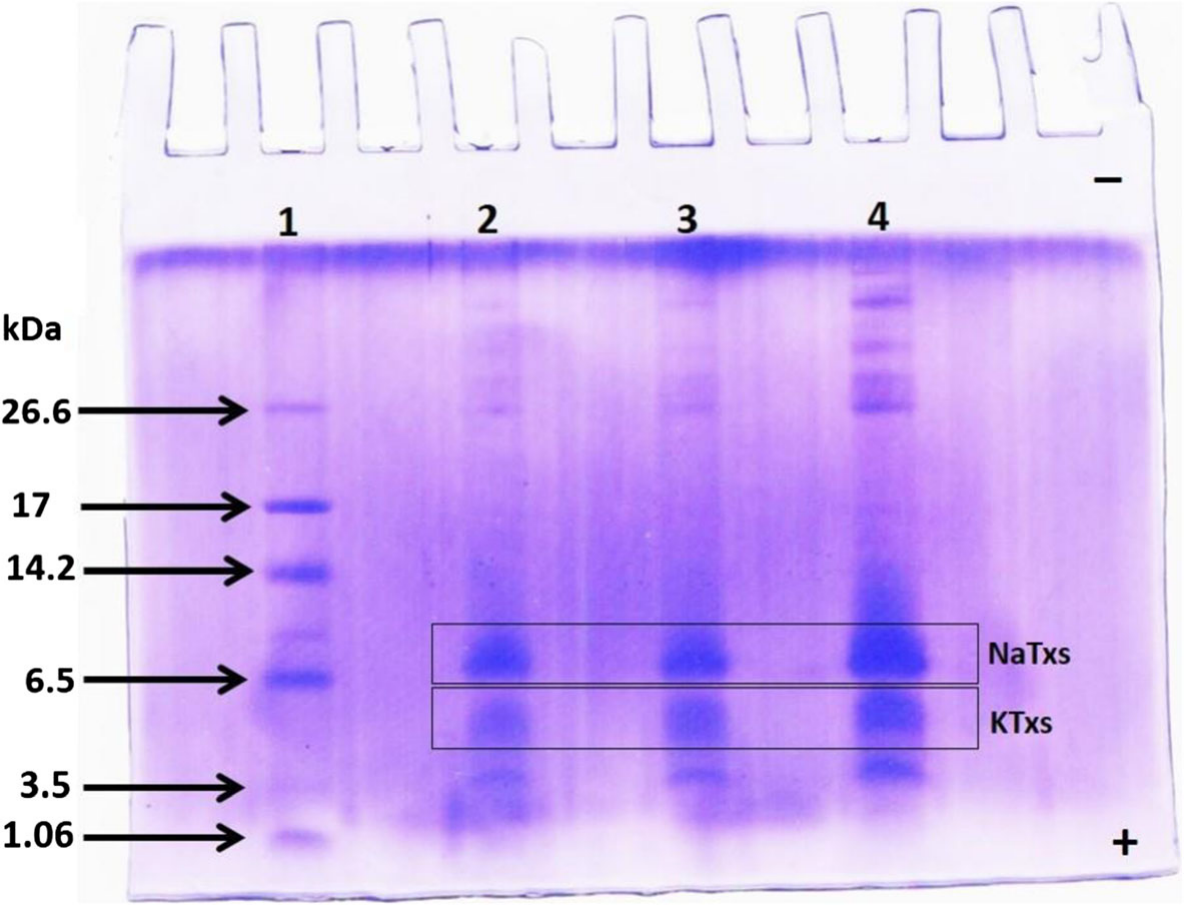
Electrophoretic profile of the pooled Ts venom. Molecular mass markers (lane 1); pooled Ts venom: 10, 20 and 30 μg (lanes 2, 3 and 4, respectively). NaTxs: voltage-gated sodium channel toxins. KTxs: voltage-gated potassium channel toxins. Coomassie Blue plus one PhastGel® R-350 staining

The Tricine-SDS-PAGE indicates the Ts venom protein profile using different masses (10, 20 and 30 μg) (Fig. 1). Two main bands of low molecular masses were observed. The sodium-channel toxins (NaTxs; from 60 to 76 amino acid residues) are the main proteins evidenced in the electrophoretic band of molecular mass between 6000 and 8000 Da, whereas the potassium-channel toxins (KTxs; from 22 to 47 amino acid residues) are the main components of the electrophoretic band from 5000 to 4000 Da.

### *T. serrulatus* venom increased the glucose levels of mice

**Fig. 2:**
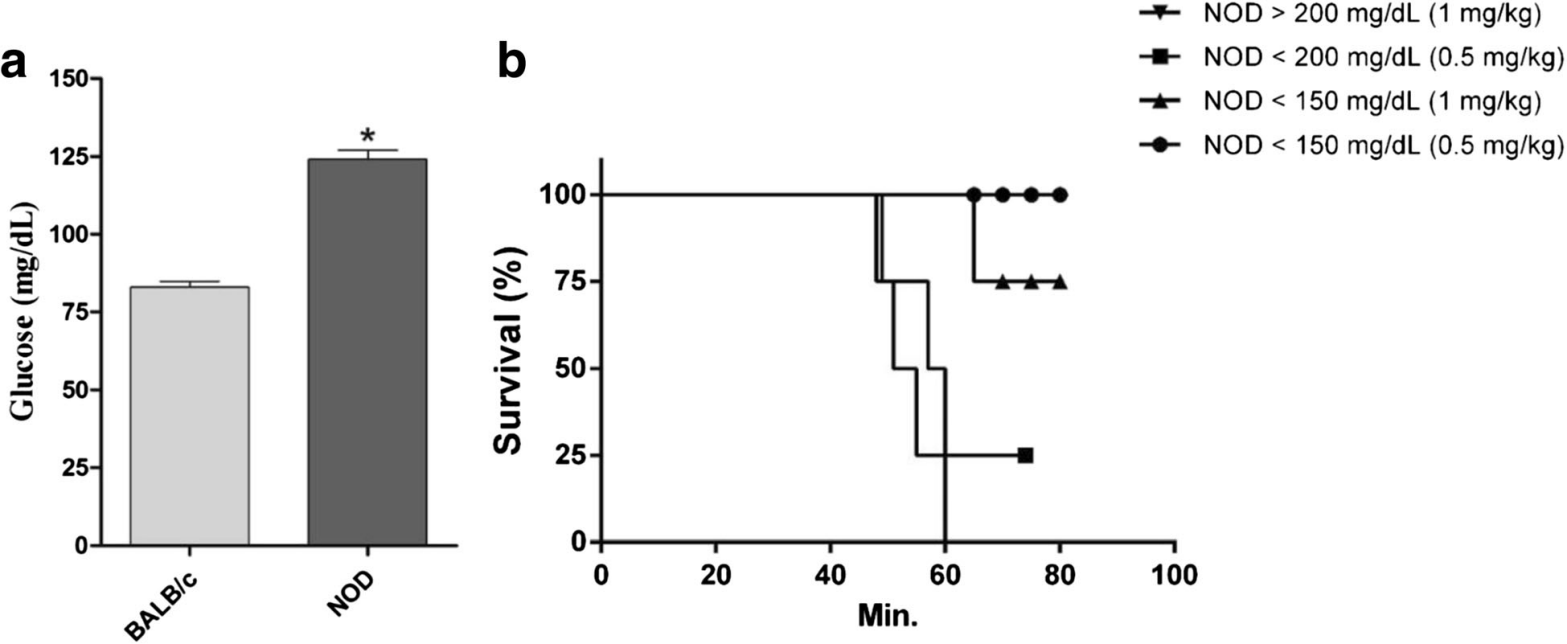
Standardization of basal glucose levels and *T. serrulatus* venom dose. **a** Basal glucose (time 0, before TsV injection) was measured in tail blood extracted from mice using a glucose meter (One Touch Ultra®, Lifescan, USA). Results are expressed as means ± SD (*n* = 4), which were analyzed by paired *t* test (*p < 0.001). **b** NOD survival using different glucose basal levels (> 200, < 200 and < 150 mg/dL) and *T. serrulatus* venom (TsV) concentrations (0.5 and 1 mg/kg)

The median basal glucose – time 0, before Ts venom (TsV) injection – was 82.9 and 125 mg/dL for BALB/c and NOD mice, respectively (Fig. 2a). Ideally, NOD mice are considered diabetic with glucose levels higher than 200 mg/dL. However, during the experimental design standardization using NOD mice, we observed that both the dose of Ts venom and the glucose basal levels were limiting factors to the experiment, inducing lethality of 25 to 100 % (Fig. 2b). Therefore, we decided to use NOD animals presenting glucose levels lower than 150 mg/dL, but significantly higher than glucose levels of the BALB/c control (*p* < 0.001) and the Ts venom concentration of 0.5 mg/kg (100 % survival).

**Fig. 3:**
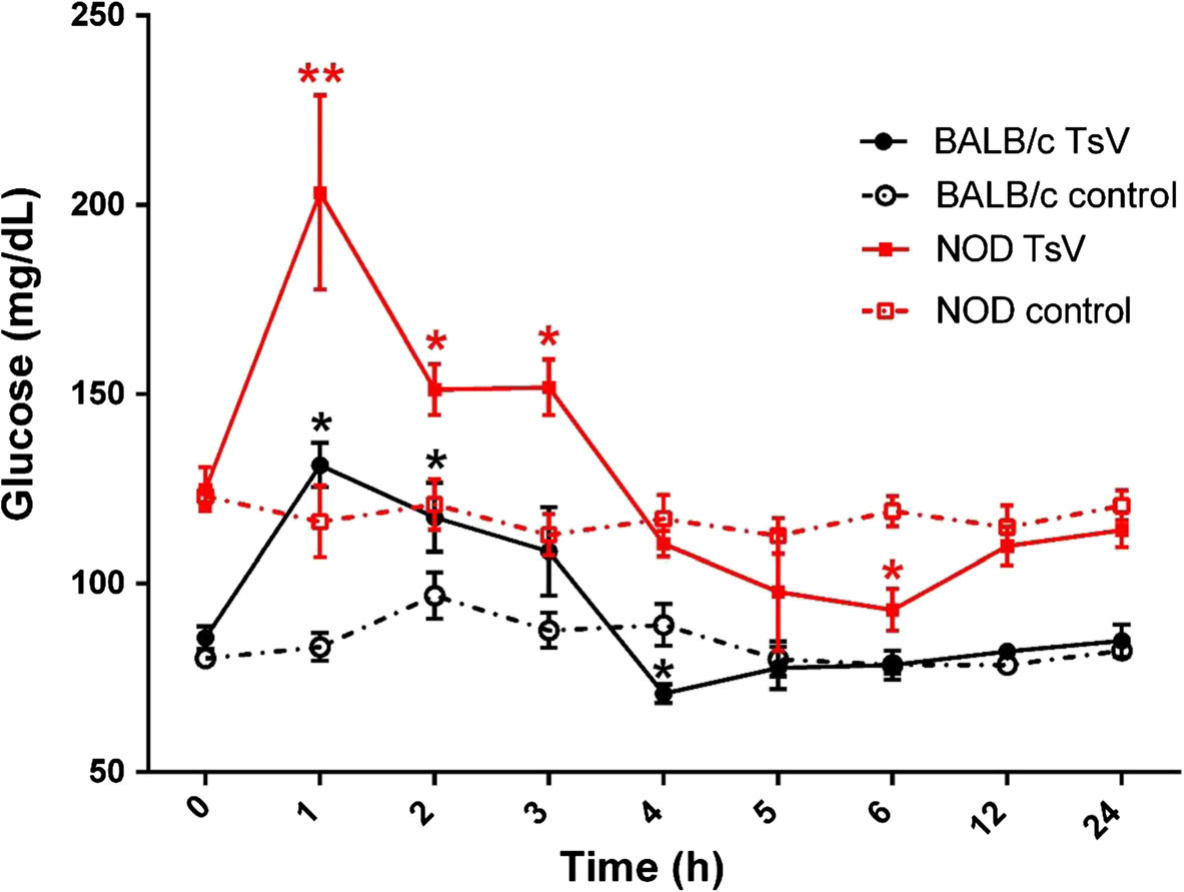
Kinetic glucose level assay of BALB/c and NOD mice injected with Ts venom. Groups of mice were injected with 0.5 mg/kg of *T. serrulatus* venom (TsV) and glucose levels were measured throughout 24 h. Glucose was measured in mouse tail blood using a glucose meter (One Touch Ultra®, Lifescan, USA). *p < 0.05 and **p < 0.001 compared to the corresponding control group. Glucose levels were significant different at all points between control groups (BALB/c control *vs* NOD control). Data are presented as means ± SD (*n* = 4), which were analyzed by ANOVA and Tukey’s multiple comparison test

All mouse groups that received 0.5 mg/kg of Ts venom showed hyperglycemia 1 h after envenomation, compared to the respective control (Fig. 3). However, the hyperglycemia was much more impressive in the NOD group, reaching glucose levels ≥ 200 mg/dL. During the analyzed time, glucose levels decrease, presenting a significant hypoglycemic period at 4 h for BALB/c and at 5 to 6 h for NOD animals. Nevertheless, the basal glucose levels for all challenged mice were reestablished after 12 h.

### *T. serrulatus* venom affected the glycosylated hemoglobin (HbA1c) of NOD mice

**Fig. 4:**
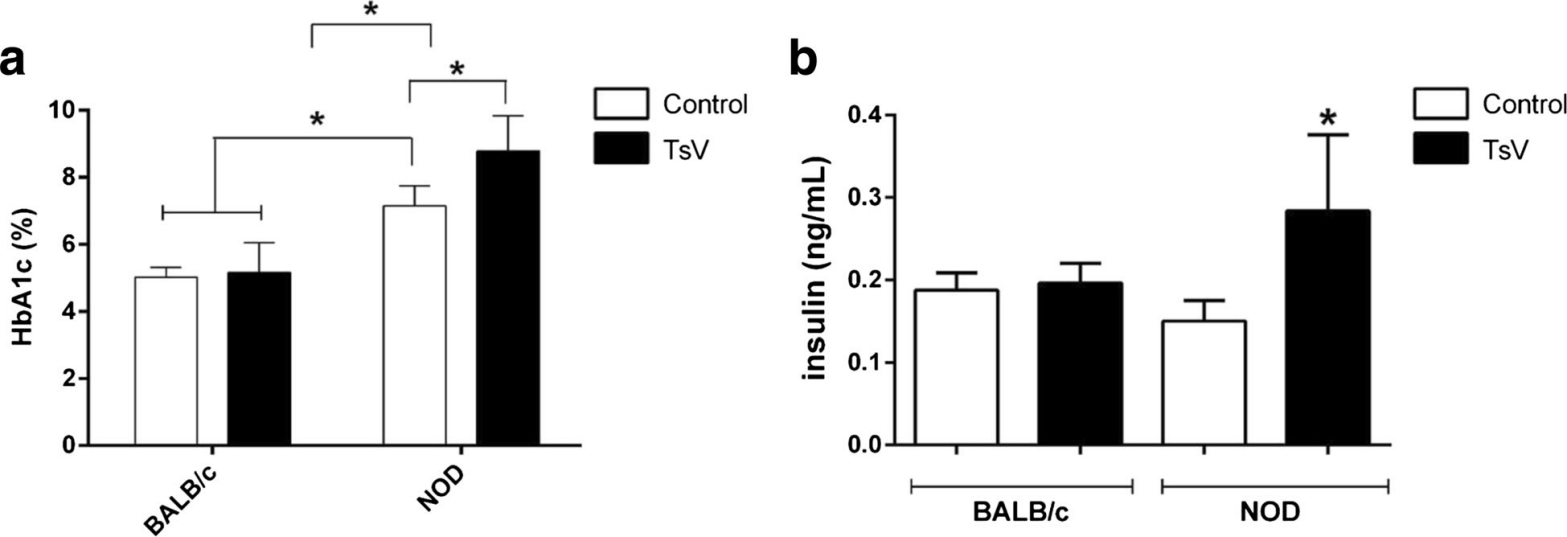
Blood glycosylated hemoglobin (HbA1c) percentage (%) and insulin plasma levels from BALB/c and NOD mice injected with Ts venom. Groups of mice were injected with 0.5 mg/kg of *T. serrulatus* venom (TsV). **a** HbA1c % was measured 30 days after challenge. **b** Insulin was measured 1 h after challenge. Results are expressed as means ± SD (*n* = 4), which were analyzed by ANOVA and Tukey’s multiple comparison test (*p < 0.05)

The total glycosylated hemoglobin (HbA1c) was analyzed in mouse blood 30 days after envenoming. The results demonstrate that although Ts venom had induced hyperglycemia in BALB/c mice, it was not able to augment HbA1c (Fig. 4a). However, Ts venom caused a significant increase of HbA1c in envenomed NOD animals. Furthermore, the NOD group control also presented a significant increase of HbA1, indicating the diabetic condition of the mice.

### *T. serrulatus* venom increased insulin levels of NOD mice

The insulin levels were analyzed in mouse plasma during the peak of hyperglycemia (1 h after envenoming). The results demonstrate that Ts venom did not change insulin levels in BALB/c mice (Fig. 4b). As to the NOD group, a significant increase of insulin was observed in the envenomed mice. On the other hand, no significant changes on insulin levels were found in the NOD control group.

## Discussion

The venom of *T. serrulatus* (Ts) is widely studied especially because of its biologically active compounds including the neurotoxins, a class of peptides mostly specific to voltage-gated sodium (Nav) or potassium (Kv) channels [29]. So far, the actions of Ts neurotoxins on Nav and Kv channels have demonstrated a gamut of physiological responses, including the increasing of plasma glucose levels [14, 15, 30–40]. Although the mechanism of hyperglycemia induced by scorpions’ venom is not clearly understood, there are studies demonstrating that it can occur through the excessive release of catecholamine, increases in glucagon and cortisol, and alterations in thyroid hormone levels or insulin secretion [41–43]. Furthermore, an isolated α-toxin from Ts, denominated Ts5, demonstrated a direct effect on isolated islets of Langerhans (not provided by catecholamine’s action), enhancing β-cell membrane depolarization and significantly potentiating glucose-induced insulin secretion [43]. Therefore, this pioneering study aimed to elucidate the effect of hyperglycemia induced by Ts venom in diabetic individuals, using a NOD mouse model.

The Ts pooled venom used in the study showed a protein profile similar to others previous described, presenting protein masses corresponding to neurotoxins specific to sodium (NaTxs) and potassium (KTxs) channels [27, 44].

The in vivo assays demonstrated that blood glucose levels significantly increase 1 h after Ts injection (0.5 mg/kg), independently of the mouse model. Indeed, the highest glucose level at 1 h after envenoming has already been described in Wistar rats using the same dose (0.5 mg/kg) and intraperitoneal (i.p.) injection, and in BALB/c mice using 1 mg/kg of Ts venom and subcutaneous (s.c.) injection [14, 15]. A peculiar observation during our experimental design standardization was that either the highest dose of Ts venom (1 mg/kg) or the highest glucose basal levels (≥200 mg/dL) cause NOD mice lethality. Furthermore, the surviving mouse that presented basal glucose ≥ 200 mg/dL and received 0.5 mg/kg of Ts venom (*n* = 1) demonstrated a clinical sequela represented by an ocular disease with partial loss of vision, probably retinopathy (Additional file 1).

The retina is an insulin-sensitive tissue and excess glucose or lipids may exert their noxious effects, accelerating retinal cell death [45]. NOD mice frequently present diabetic retinopathy, with histological analysis showing loss of retinal microvessels and reduced perfusion of the retina, which result in hypoxia with evidence of disordered focal proliferation of new vessels [46]. Definitely, diabetic retinopathy is a frequent cause of blindness developed by diabetic individuals after macular edema [47]. Nevertheless, the retinopathy induced by Ts venom in diabetic mice requires further investigation.

During the Ts venom-induced glucose kinetic assay, we also observed a period in which the glucose reached levels lower than basal glycemia, which was previously described as a hypoglycemic period [14, 15]. BALB/c mice reduced glucose levels at 4 h following Ts venom injection, while NOD demonstrated a prolonged decrease of glucose levels at 5 to 6 h following Ts venom injection. It is known that beta cells of the islets of Langerhans sense changes in the plasma glucose levels and adjust the rate of insulin production aiming to maintain the homeostatic glucose plasma concentration [48]. Thus, we assume that the production of insulin to control hyperglycemia induced by such envenoming remains high and, along with a decrease in glycogenolysis (due to hepatic glycogen depletion), causes a reduction of glucose levels.

Regarding NOD animals, the delayed hypoglycemia can be explained by the higher glucose levels required to be controlled, and the prolonged hypoglycemia (2 h) by the higher rates of insulin production. Indeed, a significant increasing of insulin was observed in NOD animals challenged with Ts venom. Although the increasing of this hormone in NOD mice seems peculiar, we assume that their diabetic disease was not sufficiently advanced to impair the insulin production significantly (the decrease of basal insulin of NOD control animals was not statistically significant compared to BALB/c control). In this sense, the animals still present a number of suitable insulin-producing beta cells in the islets of Langerhans.

According to the literature, the NOD model acquires insulin deficit after the age of 14 weeks (with high variety among individuals), that is, before this period, despite presenting high glucose levels, the animals still produce insulin. On the other hand, when these mice become overtly diabetic, they quickly lose weight and require insulin treatment [49]. The effect of high insulin levels observed in NOD-envenomed mice is somewhat controversial. This effect may be beneficial, since insulin treatment in scorpion sting victims is known as a metabolic support, which controls the adverse metabolic response produced by catecholamines and other counter-regulatory hormones [50]; or it could be unfavorable, since the injection of insulin after Ts envenoming can enhance the venom’s lethality [15]. Based on the latter, the lethality induced by Ts sting among diabetic individuals should be higher than that in healthy humans. In any case, insulin therapy after Ts envenoming should be further investigated in diabetic individuals, especially insulin-dependent ones.

On the other hand, BALB/c envenomed group did not present differences in insulin production compared to BALB/c control, although hyperinsulinemia has been reported previously during envenoming by the scorpion *Mesobuthus tamulus concanesis* and even by *T. serrulatus* [15, 42]. However, in the Ts study, the authors used a rat model and i.p. injection, which may justify such differences.

We also evaluated the glycosylated hemoglobin (HbA1c) percentage, which is the primary method recommended before initiating therapy in diabetes patients [51]. Our results demonstrated that NOD control animals surely presented a diabetic clinical condition showing HbA1c higher than 7 % while the NOD envenomed group displayed a significant elevation, with levels higher than 8 %. The augmentation of HbA1c is considered a risk factor for diabetic neuropathy and retinopathy [52]. This also explains why we observed only an ocular alteration in the envenomed group of NOD mice.

Currently, the treatment used for Ts accidents varies according to the clinical severity, which depends on the signs and symptoms manifested by the patient. Mild and moderate cases of Ts envenoming consist mostly of pain relief through analgesics at the sting site, orally or parenterally. On the other hand, severe scorpion envenoming cases require the mandatory use of the specific antivenom. In Brazil, the available antivenoms used for Ts envenoming are the scorpion antivenom (SAE or *soro antiescorpiônico* in Portuguese) and the arachnid antivenom (SAAr or *soro antiaracnídeo* in Portuguese). Their use is also compulsory in children under 7 years and in adults with previous health problems (e.g. hypertension and cardiovascular problems) even if they present mild or moderate clinical manifestations [1, 53]. In this regard, our results support the hypothesis that diabetic victims present a higher risk of developing severe envenoming. Therefore, we also advise consideration of the use of antivenom in cases of Ts envenoming in diabetic persons ─ a risk group for Ts envenoming severity.

## Conclusion

Our study, for the first time, experimentally demonstrates that accidents caused by Ts scorpion in diabetic patients should be always considered a severe case of envenoming and that greater attention should be given to these cases. Moreover, as well as the envenomed NOD animals, these patients can present a higher risk of death and sequelae. We suggest that glucose, insulin and HbA1c levels need to be carefully monitored in diabetic patients.

## Additional file

Additional file 1: Representative comparison of eyeball of Ts envenomed and non-envenomed mice. Mice were injected with Ts venom (1 mg/kg). (A) Envenomed NOD mice with glucose basal level ≥ 200 mg/dL, retinopathy indication. (B) Envenomed BALB/c mice, typical ptosis. (C) BALB/c mice control (non-envenomed), normal eyeball. (PPTX 81 kb)

## Abbreviations

DM: Diabetes mellitus
HbA1c: Glycosylated hemoglobin
i.p.: Intraperitoneal injection
KTx: Neurotoxin specific potassium channels
Kv: Voltage-gated potassium channel
NaTx: Neurotoxin specific sodium channels
Nav: Voltage-gated sodium channel
NOD: Non-obese diabetic
SDS: Sodium dodecyl sulfate
Ts: *Tityus serrulatus*
