# Supplementary figures and images for: Tityus serrulatus envenoming in non-obese diabetic mice: a risk factor for severity
Source: J Venom Anim Toxins Incl Trop Dis. 2016 Sep 17;22:26. doi: 10.1186/s40409-016-0081-8 (PMC5027101; doi:10.1186/s40409-016-0081-8)

## Slide 1
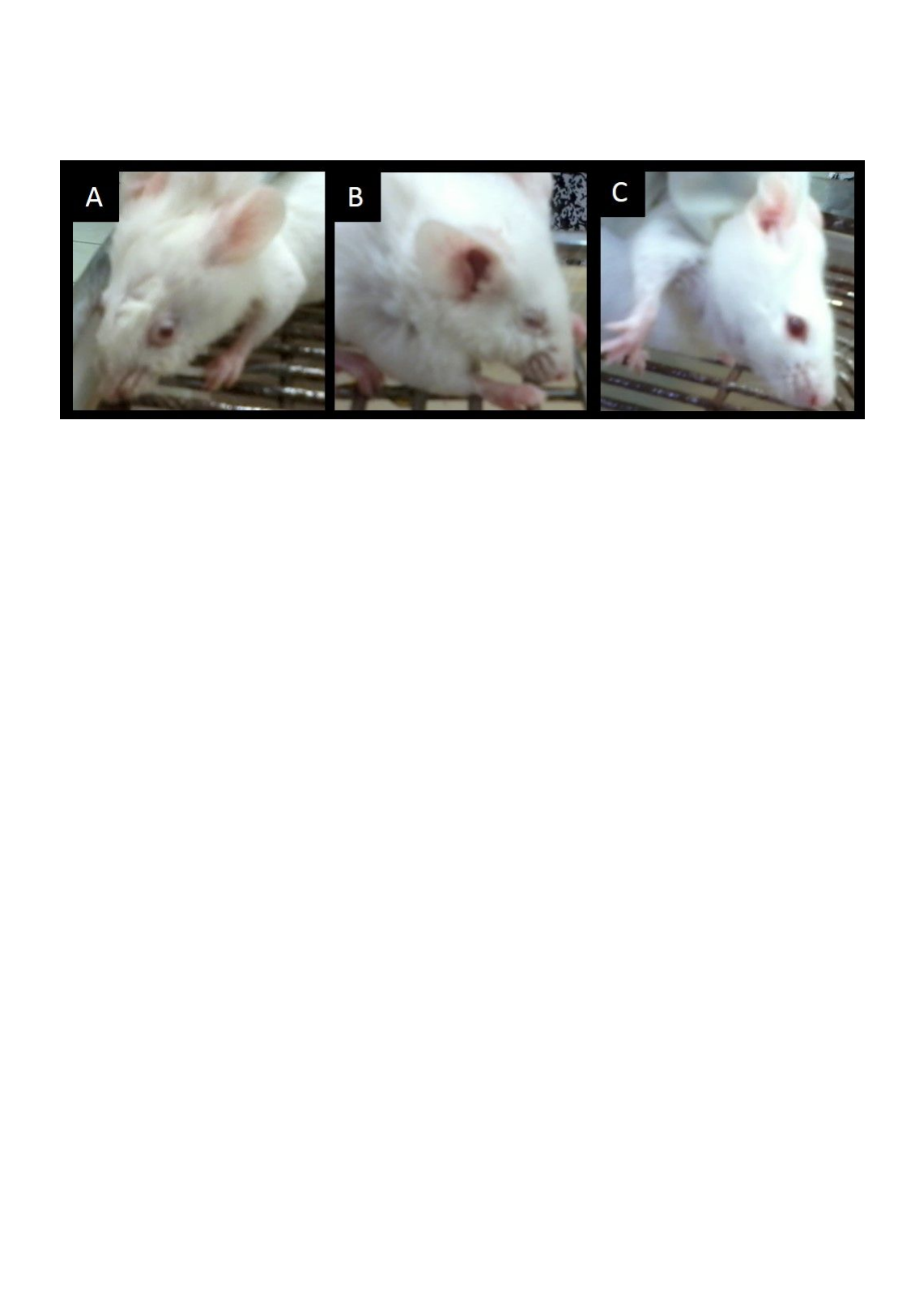

Supplement: Additional file 1: — Representative comparison of eyeball of Ts envenomed and non-envenomed mice. Mice were injected with Ts venom (1 mg/kg). (A) Envenomed NOD mice with glucose basal level ≥ 200 mg/dL, retinopathy indication. (B) Envenomed BALB/c mice, typical ptosis. (C) BALB/c mice control (non-envenomed), normal eyeball. (PPTX 81 kb) [file 40409_2016_81_MOESM1_ESM.pptx]
